# Biodistribution of Liposome-Encapsulated Bacteriophages and Their Transcytosis During Oral Phage Therapy

**DOI:** 10.3389/fmicb.2019.00689

**Published:** 2019-04-04

**Authors:** Jennifer Otero, Alba García-Rodríguez, Mary Cano-Sarabia, Daniel Maspoch, Ricard Marcos, Pilar Cortés, Montserrat Llagostera

**Affiliations:** ^1^Departament de Genèticai de Microbiologia, Universitat Autònoma de Barcelona, Barcelona, Spain; ^2^Catalan Institute of Nanoscience and Nanotechnology (ICN2), CSIC and the Barcelona Institute of Science and Technology, Barcelona, Spain; ^3^Institució Catalana de Recerca i Estudis Avançats (ICREA), Barcelona, Spain; ^4^Consortium for Biomedical Research in Epidemiology and Public Health (CIBERESP), Carlos III Health Institute, Madrid, Spain

**Keywords:** biodistribution, transcytosis, liposomes, bacteriophages, phage therapy

## Abstract

This study sheds light on the biodistribution of orally administered, liposome-encapsulated bacteriophages, and their transcytosis through intestinal cell layers. Fluorochrome-labeled bacteriophages were used together with a non-invasive imaging methodology in the *in vivo* visualization of bacteriophages in the stomach and intestinal tract of mice. In those studies, phage encapsulation resulted in a significant increase of the labeled phages in the mouse stomach, even 6 h after their oral administration, and without a decrease in their concentration. By contrast, the visualization of encapsulated and non-encapsulated phages in the intestine were similar. Our *in vivo* observations were corroborated by culture methods and *ex vivo* experiments, which also showed that the percentage of encapsulated phages in the stomach remained constant (50%) compared to the amount of initially administered product. However, the use of conventional microbiological methods, which employ bile salts to break down liposomes, prevented the detection of encapsulated phages in the intestine. The *ex vivo* data showed a higher concentration of non-encapsulated than encapsulated phages in liver, kidney, and even muscle up to 6 h post-administration. Encapsulated bacteriophages were able to reach the liver, spleen, and muscle, with values of 38% ± 6.3%, 68% ± 8.6%, and 47% ± 7.4%, respectively, which persisted over the course of the experiment. Confocal laser scanning microscopy of an *in vitro* co-culture of human Caco-2/HT29/Raji-B cells revealed that Vybrant-Dil-stained liposomes containing labeled bacteriophages were preferably embedded in cell membranes. No transcytosis of encapsulated phages was detected in this *in vitro* model, whereas SYBR-gold-labeled non-encapsulated bacteriophages were able to cross the membrane. Our work demonstrates the prolonged persistence of liposome-encapsulated phages in the stomach and their adherence to the intestinal membrane. These observations could explain the greater long-term efficacy of phage therapy using liposome-encapsulated phages.

## Introduction

With the increasingly frequent appearance and expansion of multidrug-resistant bacteria, there has been a re-evaluation of the therapeutic use of bacteriophages. The advantages of lytic phages over traditional antimicrobials include the ability of phages to self-replicate and their high specificity toward the target bacterium, without affecting the host microbiota. Furthermore, lytic phages do not cause human allergies, nor do they change the structure, odor or flavor of food products (Hagens and Offerhaus, 2008). These characteristics make bacteriophages of interest for use in veterinary and human medicine, as well as in the food industry (Vandenheuvel et al., 2015; Young and Gill, 2015; Cooper et al., 2016; Górski et al., 2016; Pelfrene et al., 2016; Abedon et al., 2017; Cortés and Llagostera, 2017).

Among the different routes of bacteriophage administration, the oral route is likely to be the most appropriate for phage therapy in humans and animals (Zelasko et al., 2017). Its advantages compared to other routes of parenteral administration include the relative ease delivery, a potentially low immunogenicity, and greater patient comfort, among others (Zelasko et al., 2017). However, oral phage therapy poses several challenges. Phages typically lack stability in the extremely acidic environment of the stomach (Jonczyk et al., 2011), and their residence time in the intestine is very short. In a previous study, we tested a method aimed at overcoming these potential drawbacks, by examining the efficacy of phages encapsulated in cationic lipid envelopes (liposomes) (Colom et al., 2015). The resulting phage-containing nanocapsules (mean diameter of 309–326 nm) were significantly more stable than non-encapsulated bacteriophages in simulated gastric fluid (pH 2.8). In addition, in broiler chickens, the administration of liposome-encapsulated bacteriophages resulted in phage detection after 72 h in 38.1% of the treated animals. By contrast, only 9.5% of the chickens retained the non-encapsulated bacteriophages. We also demonstrated the enhanced efficacy of liposome-encapsulated phage therapy in the prolonged protection of poultry against *Salmonella* (Colom et al., 2015). Based on those results, we hypothesized that the liposomes could adhere to the intestinal epithelium and the encapsulated bacteriophages could undergo transcytosis. However, we found no published studies reporting either of these phenomena, although the adherence of non-encapsulated bacteriophages to the intestinal barrier, their diffusion across the mucosal surface, and their translocation through intestinal cells have been described (Barr et al., 2015; Barr, 2017; Shan et al., 2018). Other studies demonstrated the capacity of non-encapsulated bacteriophages to migrate into the blood and enter internal organs (Dabrowska et al., 2005; Górski et al., 2006; Hamzeh-Mivehroud et al., 2008; Oliveira et al., 2009; Górski et al., 2016).

Thus, in the present work we studied the adherence of liposome-encapsulated phages to the intestinal barrier and the ability of those phages to cross the barrier. Specifically, we investigated the *in vivo* and *ex vivo* biodistribution of orally administered, fluorochrome-stained, liposome-encapsulated bacteriophages using a non-invasive imaging methodology. In addition, the presence of bacteriophages in *ex vivo* organs was determined using culture methods. Phage transcytosis was examined using an *in vitro* model of the human intestinal barrier, consisting of a triple co-culture of Caco-2 and HT29 intestinal cells with Raji-B lymphocytes.

## Materials and Methods

### Bacterial Strains and Bacteriophage Lysates

*Salmonella enterica* serovar Typhimurium LB5000 (SGSC181; University of Calgary) was grown in Luria-Bertani (broth with shaking or on Luria-Bertani agar plates for 18 h at 37°C and was used to propagate and quantify bacteriophage UAB_Phi20. This bacteriophage, a member of the *Podoviridae* family, has an icosahedral head and non-contractile tail (60 ± 2.7 nm and 13 ± 0.7 nm, respectively) (Bardina et al., 2012, 2016). Its genome has been fully sequenced (Bardina et al., 2016). Phage lysates were prepared following a previously described method (Colom et al., 2015) and filtered through 0.45-μm and 0.22-μm-pore-size polyethersulfone (PES) membranes (Millex^®^-HP; RRID:SCR_008983), followed by ultracentrifugation at 68.584 × *g* for 2 h (90Ti rotor; OptimaTM × PN-100, Beckman coulter, RRID:SCR_008940). The resulting lysate was additionally purified using an Amicon filtration device (Ultra-15 Centrifugal 100 kDa MWCO; Millipore, RRID:SCR_008983) and centrifugation at 5,000 × *g* for 15 min (Eppendorf 5810R; Eppendorf, RRID:SCR_000786). The phages were then washed several times with 10 mM MgSO_4_ under the same conditions. The final concentration of the purified phage lysate was 1–3 × 10^12^ pfu/mL.

### Cell Lines and Culture Conditions

The human colorectal adenocarcinoma cell lines Caco-2 HT29 and the human B lymphocyte line Raji were used in a co-culture to mimic the intestinal barrier. Caco-2 cells were kindly provided by Dr. Isabella Angelis (Istituto Superiore di Sanità, Italy), HT29 (ATCC^®^ HTB-38^TM^) and the Raji-B cell line (ATCC^®^ CCL-86^TM^) were purchased from the American Type Culture Collection (ATCC, RRID:SCR_001672). All cell lines were maintained in Dulbecco’s modified Eagle’s high-glucose medium without pyruvate (DMEM w/o pyruvate; Life Technologies, RRID:SCR_008817). The medium was supplemented with 10% fetal bovine serum (FBS), 1% non-essential amino acids (NEAA; PAA Laboratories GmbH, Pasching, Austria) and 2.5 mg plasmocin (InvivoGen, San Diego, CA, United States)/mL. The cell cultures were incubated at 37°C in a humidified atmosphere of 5% CO_2_ and 95% air. Routinely, the Caco-2 and HT29 cell lines were sub-cultured once a week with 1% trypsin-EDTA (PAA Laboratories GmbH) at 7.5 × 10^5^ cells/75 cm^2^ flask and 4 × 10^5^ cells/75 cm^2^ flask, respectively.

### Phage Encapsulation

Liposome encapsulation of purified UAB_Phi20 (1 × 10^11^ pfu/mL) was carried out using the film-hydration method. The lipid mixture consisted of 1,2-dilauroyl-rac-glycero-3-phosphocholine (DLPC), cholesteryl polyethylene glycol 600 sebacate (Chol-PEG600), cholesterol (Chol), and cholesteryl 3β-N (dimethylaminoethyl) carbamate hydrochloride (cholesteryl) in a molar ratio of 1:0.1:0.2:0.7. The mixing process was previously described (Colom et al., 2015; Cortés et al., 2018). The resulting suspension of large multilamellar vesicles was homogenized using an extruder (Lipex Biomembranes, Vancouver, Canada) and a polycarbonate membrane (pore size, 400 nm) to obtain unilamellar vesicles. The particle-size distributions and zeta potential of the nanocapsules were determined in a ZetaSizer Nano ZS apparatus, by measuring the electrophoretic mobility and using a dynamic light scattering (DLS) analyzer combined with non-invasive backscatter technology (Colom et al., 2015; Cortés et al., 2018). One mL of each sample was measured without dilution. The mean diameter was the median of three different measurements.

The percentage of bacteriophages encapsulated by this method was calculated as follows: percentage (%) = 100-(C_free_/C_total_)-100 (Colom et al., 2015), where C_free_ is the titration of appropriate dilutions of the encapsulation mixture directly onto strain LB5000 agar plates using the double agar layer method. The total phage concentration (C_total_) was determined by mixing 0.5 ml of the liposome-phage dilutions with 0.5 ml (50 mM) of bile salts (Sigma-Aldrich, RRID:SCR_008988) to disrupt the liposomes and then plating the resulting suspensions on agar plates using the double agar layer method. The encapsulation efficiency was obtained from three independent encapsulation experiments, and in each experiment, the values come from triplicate plates of each dilution.

### Fluorescence Labeling of the UAB_Phi20 Bacteriophage

Purified UAB_Phi20 bacteriophage (1 × 10^12^ pfu/mL) was stained with the fluorochrome Vivo-Tag S 750 (PerkinElmer, MA, United States) by adding 100 μl of fluorochrome per 1 mL of phage, followed by a 1-h incubation at room temperature in the dark. The bacteriophages were then diluted 10-fold in MgSO_4_ 10 mM and washed by ultrafiltration using an Amicon Ultracel 10 KDa (Millipore, RRID:SCR_008983) filtration unit at 5000 × g at 20°C for 5 min. The fluorochrome:bacteriophage ratio was calculated by measuring the absorbance at 750 and 280 nm using a Nanodrop 2000 spectrophotometer (Thermo Fisher Scientific, RRID:SCR_008452) according the manufacturer’s instructions. The stained-bacteriophage concentration was also calculated by titration with test strain LB5000. Stained bacteriophage was encapsulated in the liposome mixture as described for the *in vivo* distribution study.

To measure *in vitro* fluorescent light production by the non-encapsulated and liposome-encapsulated labeled bacteriophage, serial half-dilutions ranging from 5.8 × 10^11^ to 1.1 × 10^9^ pfu/mL were prepared in a 96-well black plate. The fluorescence signal (FLI) was qualitatively evaluated as the radiant efficiency (RE, fluorescence emission radiance per incident excitation power) and the values for each well were plotted with respect to the phage concentration. VTS-750 fluorescence imaging was carried out using the IVIS Spectrum imaging system (PerkinElmer RRID:SCR_012163), and the images and RE signal were registered and analyzed using Living Image 4.5 software (PerkinElmer RRID:SCR_012163). The mean FLI intensity, and corresponding standard error of the mean (SEM) or standard deviation (SD) were determined. All analyses and graph construction were performed using GraphPad Prism 5 software (GraphPad Prism, RRID:SCR_002798).

### *In vivo* Biodistribution of Bacteriophage in Murine Model

The biodistribution of liposome-encapsulated and non-encapsulated labeled UAB_Phi20 bacteriophage was evaluated *in vivo* and *ex vivo* in 5-week-old athymic nude female mice (*Mus musculus*, strain Hsd:Athymic Nude-Foxn1; ENVIGO, Santa Perpètua de la Mogoda, Spain). The mice were housed in quarantine rooms during an acclimatization period of 8 days and inspected by a veterinarian, who confirmed the health of the mice. The mice were then randomly housed under specific-pathogen-free (SPF) conditions in autoventilated racks. Food and water were supplied *ad libitum*.

Both liposome-encapsulated and non-encapsulated stained bacteriophages were orally administered by oral gavage of a single dose of 1.3 × 10^13^ pfu/kg mouse body weight; the dosing volume was 22.4 mL/kg. For each group (8 animals), a non-treated mouse was included as an autofluorescence control of background tissue-fluorescence levels. At 2.5 and 5.5 h post-administration, the whole-body biodistribution of the bacteriophages was measured non-invasively in the mice by VTS-750 fluorescence image monitoring, acquiring ventral and dorsal views. In addition, 30 min later, at 3 h and 6 h after phage administration, stomach, intestine, spleen, liver, kidney, muscle, and blood samples were collected from four animals and bacteriophage accumulation in the respective tissues was determined by *ex vivo* VTS-750 fluorescence monitoring, acquiring ventral, and dorsal views of the organs. Thereafter, all tissues were transferred to MgSO_4_ buffer (10 mM). The animals were euthanized by cervical dislocation while still anesthetized, following standard procedures for euthanasia.

The bacteriophage concentration was determined in whole organs (stomach, intestine, liver, spleen, and kidney), a dorsal portion of muscle and the volume of blood sampled by cardiac puncture to obtain plasma. All samples were weighed and then resuspended in 2 mL of MgSO_4_ (10 mM). Afterward, the samples were mechanically homogenized for 15 min and serial dilutions of the homogenates were plated using the double agar layer method. The concentration of encapsulated bacteriophages was also determined in the tissues of mice treated with liposome-encapsulated phages, following the procedure described above. In addition, the presence or absence of bacteriophages was checked in all tissues, including those of control mice not treated with bacteriophages, using enrichment protocols (Guttman et al., 2005).

*In vivo* and *ex vivo* FLI was determined using the IVIS Spectrum imaging system (PerkinElmer RRID:SCR_012163), as described above. *Ex vivo* FLI values were calculated by measuring the RE of both the dorsal and ventral view of each organ and then considering the weight of each organ, transforming the RE values to the RE per gram of tissue.

The *in vivo* experiments were performed using ICTS “NANBIOSIS,” specifically, the CIBER-BBN *in vivo* experimental platform of the Functional Validation & Preclinical Research (FVPR) area^1^ and Laboratory Animal Service (LAS) of Vall d’Hebron Institut de Recerca (VHIR; Barcelona). The animals were treated in compliance with the guidelines of the Ethics Commission (Comité Ético de Experimentación Animal [CEEA]) of the VHIR, Barcelona. The study was approved and assigned the authorization number 69/15.

### Phage Transcytosis in *in vitro* Model of Intestinal Barrier

Bacteriophage transcytosis was assayed on Caco-2 and HT29 cells seeded in 12-well culture plates using a polyethylene terephthalate Transwell^®^ (PET) with a 1-μm pore size and an area of 1.12 cm^2^ (Merck Millipore, Darmstadt, Germany) as a support chamber to establish apical and basolateral sides. Briefly, 1.7 × 10^5^ Caco-2 and HT29 cells clones were mixed in DMEM with FBS and seeded onto the apical compartment of the Transwell at a cell ratio of 90:10, respectively. On day 14 after seeding, Raji-B lymphocytes were added to the basolateral side of the Transwell to promote differentiation of the Caco-2 cells to M-cells. The co-cultures were left to differentiate for 21 days, until a monolayer of polarized cells had formed. The culture medium was changed every 2 days (García-Rodríguez et al., 2018).

After 21 days of incubation, the transepithelial electrical resistance (TEER) of the cell monolayers was measured using an ohmmeter (Millicell ERS-2 Voltohmmeter; Merck Millipore, Darmstadt, Germany). Only values >300 Ω/cm^2^ were accepted in the analysis. UAB_Phi20 bacteriophage (0.5 mL, 1 × 10^10^ and 1 × 10^7^ pfu/mL) in DMEM with FBS medium was inoculated on the apical side of the Transwell. At 2, 6, 24, and 48 h post-inoculation, bacteriophages from the apical and basolateral sides of the Transwell were quantified on lawns of strain LB5000, as previously described. Accordingly, the basolateral volume (1.5 mL) was removed and replaced with fresh FBS-containing DMEM. The TEER was measured after each transcytosis test to ensure the integrity and polarization of the cell cultures. Liposome-encapsulated UAB_Phi20 bacteriophage (5 × 10^9^ pfu/well) was assayed at the same time points and under the same conditions but in DMEM without FBS because the presence of FBS resulted in aggregation of the liposomes.

### Transmission Electron Microscopy (TEM)

Liposome-encapsulated phage was examined by cryogenic transmission electron microscopy (cryo-TEM) using a JEOL JEM-1400 microscope (JEOL, Japan). The samples were prepared as detailed in previous publications (Colom et al., 2015; Cortés et al., 2018).

Cell monolayers incubated with bacteriophages for 48 h within the apical compartment of the Transwell were aspirated, washed with phosphate-buffered saline (PBS 1 X), and fixed with 2.5% glutaraldehyde in 0.1 M cacodylate buffer (pH 7.2) at 4°C for 2 h. The fixed cells were then washed four times with MiliQ-grade water and dehydrated with increasing concentrations of acetone. After the cells had been embedded in polymerized Epon 812 resin for 48 h, ultrathin sections (60–70 nm) were cut using an ultracut microtome (Leica Microsystems, RRID:SCR_008960) and counterstained first with uranyl acetate for 30 min and then with Reynolds solution lead citrate for 5 min. The cell monolayers were observed in a JEOL JEM-1400 microscope (JEOL, Japan).

### Confocal Laser Scanning Microscopy (CLSM)

Bacteriophage and liposomes were stained with SYBR Gold and Vybrant DiI (Thermo Fisher Scientific, MA, United States), respectively, as previously described (Colom et al., 2015). Confirmation of the liposome encapsulation of the phages was carried out by CLSM using a Leica TCS SP5 confocal microscope (Leica Microsystems, RRID:SCR_008960). In this case, the liposomes were not extruded through polycarbonate membrane, as the final particles were below the resolution limit of the optical microscope (Colom et al., 2015).

In addition, both non-encapsulated and liposome-encapsulated bacteriophages (0.5 mL; 1 × 10^10^ pfu/mL) were transferred to the apical compartment of the Transwell for 2 h at 37°C in the dark. Afterward, the cell monolayers were washed and fixed with 4% paraformaldehyde (Sigma-Aldrich, MO, United States) for 15 min at 37°C, washed with PBS 1 X, and maintained overnight at 4°C. The cell nuclei and plasma membranes were then stained for 15 min at room temperature with Hoechst 33342 and CellmaskTM Deep Red plasma (Thermo Fisher Scientific, RRID:SCR_008452), respectively. After staining, the cell layers were left to stand face down in 35-mm glass-bottom culture dishes with 14-mm microwells (MatTek Corp., MA, United States). The cell monolayers were imaged on a Leica TSC SP5 confocal microscopy (Leica Microsystems, RRID:SCR_008960) using the PL APO 63 × /1.4-0.6 oil CS UV objective (Leica Microsystems, RRID:SCR_008960). The images were processed with Fiji (Schindelin et al., 2012) and IMARIS^®^ (Bitplane, Zurich, Switzerland) software to visualize the confocal stacks and to obtain three-dimensional cross-section images with merged labels.

### Statistical Analysis

The results are expressed as the mean and SD. A Kolmogorov-Smirnov test was used to check the normality of the samples, and a repeated measure two-way ANOVA and Tukey’s multiple comparisons test were performed as appropriate, using GraphPad Prism version 6.00 (GraphPad Prism, RRID:SCR_002798).

## Results

### *In vivo* Biodistribution of Bacteriophages in Mice

Data on the encapsulation of VTS-750-stained UAB_Phi20 phage indicated that the final product was a mixture containing ∼46% encapsulated and 54% non-encapsulated UAB_Phi20 phages (Supplementary Table S1). The size of the liposomes as estimated by DLS was 341.6 ± 8.6 nm with a zeta potential of + 34 ± 5.0 mV. Encapsulation was also confirmed by visualization of the nanocapsules on the cryo-TEM images and the three-dimensional (3D) spatial superimposition of the CLSM images showing the fluorescence of both the phages and liposomes (Supplementary Figure S1). The term “the product of phage encapsulation” (PPE) is used in the following to refer to this mixture.

Prior to the *in vivo* experiments, the RE of dilutions of VTS-750-stained UAB_Phi20 (5.8 × 10^11^ to 1.1 × 10^9^ pfu/mL) was measured using the IVIS^®^ Spectrum imaging system. Measurements of the same dilutions of PPE and VTS-750-stained UAB_Phi20 showed that, although a quenching effect occurred at higher bacteriophage concentrations, the correlations between fluorescence and the bacteriophage concentration were linear between 4.5 × 10^9^ and 3.6 × 10^10^ pfu/mL for UAB_Phi20 phage and between 2.3 × 10^9^ and 1.8 × 10^10^ pfu/mL for PPE. The FLI of the bacteriophage was higher (3.4 ± 1.1 times) than that of PPE, indicating that liposome encapsulation resulted in fluorescence attenuation, which was considered in subsequent *in vivo* measurements.

**FIGURE 1:**
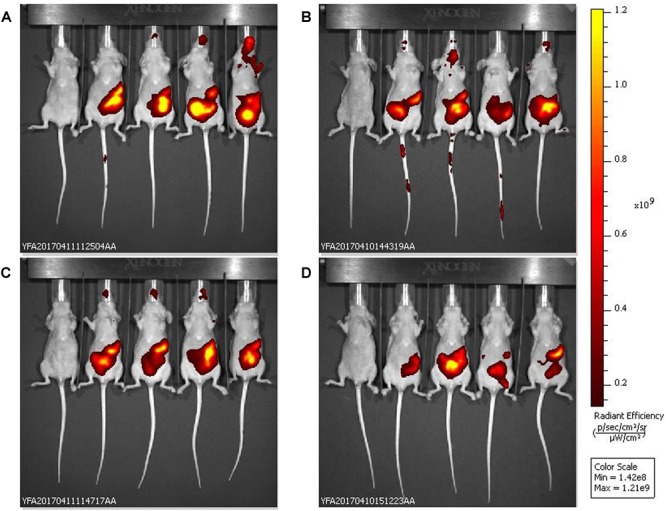
*In vivo* fluorescence images of the biodistribution in mice of VTS-750-stained UAB_Phi20: non-encapsulated phages **(A,C)** and the products of phage encapsulation (PPE) **(B,D)**. The ventral side of each mouse was imaged 2.5 h **(A,B)** and 5.5 h **(C,D)** after oral administration of the phage preparations (*n* = 4 mice per group). The pseudocolor scale bars are consistent for each corresponding view and show the relative changes over time.

In the *in vivo* experiments, single oral doses (1.3 × 10^13^ pfu/kg) of bacteriophage UAB_Phi20 and of PPE were administered to two groups of mice. Both preparations were well tolerated and did not cause significant body weight loss or adverse side effects in the mice. *In vivo* fluorescence imaging showed that, by 2.5 h post-administration, UAB_Phi20 had mainly accumulated in the gastrointestinal tract (stomach and intestinal system), decreasing slightly in these organs by 5.5 h post-administration (Figure 1). Similar results were achieved following PPE administration. In either case, the presence of phages in other tissues was not detected by *in vivo* fluorescence imaging.

**FIGURE 2:**
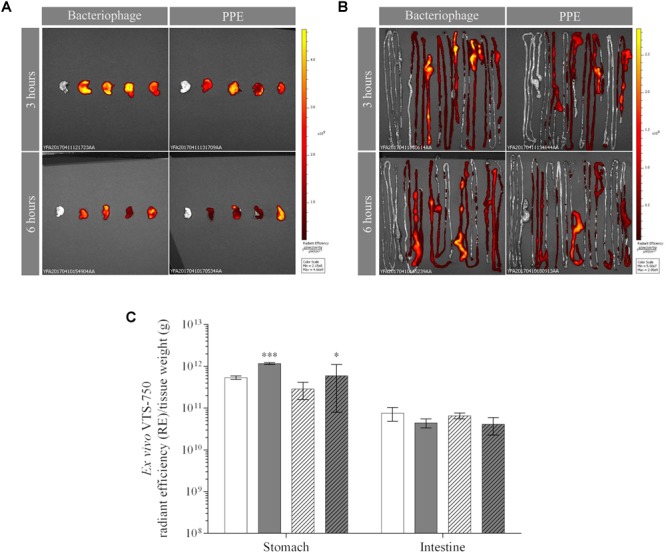
*Ex vivo* fluorescence of bacteriophages and PPE in the mouse stomach **(A)** and intestine **(B)** 3 and 6 h post-administration. The dorsal view of both organs is shown. Pseudocolor scale bars are located on the right for each corresponding set of images. **(C)** Fluorescence accumulation (RE) per tissue weight (g) in the stomach and intestine 3 h (solid) and 6 h (dotted) after the administration of non-encapsulated phages (white) or PPE (gray). Values of RE/g of PPE were obtained after taking into account the fluorescence attenuation (3.4-fold) observed in the *in vitro* experiment. The significance of the RE/g values of the encapsulated vs. non-encapsulated phage is shown: ^∗∗∗^*P* < 0.001 and ^∗^*P* < 0.05.

The *ex vivo* FLI results confirmed those obtained *in vivo*, as they revealed the accumulation of UAB_Phi20 fluorescence (RE) in the stomach (Figure 2A) and intestine (especially the cecum) in both groups of animals (Figure 2B). Calculations of the RE/g of PPE (Figure 2C) took into account the attenuation of the fluorescence observed in the *in vitro* measurements (3.4-fold). Thus, 3 and 6 h after treatment, the bacteriophages were mainly present in the stomach, and the FLI (RE/g) of PPE was significantly higher than that of the non-encapsulated phage (Figure 2C). In the intestine, FLI was maintained over time, with slightly larger numbers of bacteriophages present in mice treated with UAB_Phi20 than with PPE, both at 3 and 6 h post-administration (Figure 2C). In the spleen, liver, and kidney, as well as in muscle, the FLI in mice treated with UAB_Phi20 and PPE indicated a generally low and variable accumulation of the phages among replicates, attributable to the limit of detection of the imaging technique. Thus, at a concentration of stained bacteriophages ≤ 7 log_10_ pfu/g, the imaging methodology did not allow discrimination between the autofluorescence of the mouse tissues and the fluorescence of the stained phages (data not shown).

After the *ex vivo* FLI measurements, the tissues were weighed, homogenized in MgSO_4_ (10 mM), and the bacteriophage concentration was determined, including the concentration of encapsulated bacteriophages present in the tissues of PPE-treated mice. The latter assessment was accomplished by titration of the phages in untreated tissues and in tissues treated with bile salts, as previously described. This allowed calculation of the percentage of encapsulated phages present in each tissue (Table 1). The results in the organs and tissues harvested 3 and 6 h post-administration showed that the encapsulated phages remained or had accumulated in the spleen (68 ± 8.6% and 69 ± 7.6%, respectively), stomach (50 ± 8.2% and 53 ± 9.4%, respectively), and muscle (47 ± 7.4% and 47 ± 6.6%, respectively). The corresponding percentages in the liver were lower (38 ± 6.3% and 23 ± 1.6%). By contrast, liposome-encapsulated bacteriophages were not found in either the intestine or the kidney, and the percentage in plasma was the lowest measured (Table 1).

**Table 1:** Bacteriophage concentrations in *ex vivo* organs obtained from mice orally administered bacteriophages and the products of phage encapsulation (PPE).

Organ	Time (h)	Bacteriophage Concentration (log_10_ pfu/g) ^a^	PPE	
			Concentration (log_10_ pfu/g)^a,b^	Percentage (%) ^a^
Stomach	3	7.6 ± 0.2	10.0 ± 0.2^d^	50 ± 8.2
	6	7.4 ± 0.6	9.9 ± 0.2^d^	53 ± 9.4
Intestine	3	9.0 ± 0.2	9.2 ± 0.1	0 ± 0.0
	6	9.4 ± 0.2	9.2 ± 0.2	0 ± 0.0
Liver	3	7.2 ± 0.4	6.6 ± 0.2	38 ± 6.3
	6	7.3 ± 0.6^c^	4.8 ± 0.7	23 ± 1.6
Spleen	3	6.6 ± 0.6^c^	4.4 ± 0.1	68 ± 8.6
	6	5.4 ± 0.5	5.1 ± 0.9	69 ± 7.6
Kidney	3	7.6 ± 0.3^c^	5.7 ± 0.2	0 ± 0.0
	6	6.5 ± 0.3^c^	4.0 ± 0.1	0 ± 0.0
Muscle	3	6.1 ± 0.2	4.9 ± 0.7	47 ± 7.4
	6	6.4 ± 0.6^c^	4.6 ± 0.4	47 ± 6.6
Plasma	3	4.9 ± 0.5	3.7 ± 0.9	21 ± 9.3
	6	5.1 ± 0.1	4.9 ± 0.3	17 ± 7.2

*^*a*^Each value is the average of four samples ± standard deviation. ^*b*^Values obtained in samples treated with bile salts. ^*c*^Statistically significant difference between non-encapsulated and encapsulated phages (*P* < 0.001 at 3 h in the spleen and kidney and at 6 h in the liver and kidney; *P* < 0.05 at 6 h in muscle). ^*d*^Statistically significant difference between encapsulated and non-encapsulated phage (*P* < 0.001). The difference in the percentage of encapsulation at 3 and at 6 h was not significant.*

The total phage concentrations in the organs and tissues of the two groups of animals are shown in Table 1. Throughout the study, phage concentrations in the stomach were significantly higher in PPE-treated mice than in mice treated with non-encapsulated phages, whereas the differences in the intestinal concentrations of the two phage preparations was not significant (Table 1). In other tissues and organs, the phage concentrations were higher in mice administered non-encapsulated phages. Specifically, the non-encapsulated phage concentration in the liver at 3 and 6 h was 7.2 ± 0.4 and 7.3 ± 0.6 log_10_ pfu/g, respectively. These levels were maintained over time, whereas in the PPE-treated mice the concentration in the liver decreased from 6.6 ± 0.2 log_10_ pfu/g at 3 h to 4.8 ± 0.7 log_10_ pfu/g at 6 h. In the kidney, the accumulation of non-encapsulated phages and PPE decreased between 3 and 6 h of administration, whereas a decrease in the spleen occurred only in mice treated with the former preparation. The phage concentration increased between 3 and 6 h post-administration in the spleen of the PPE-treated mice and in the plasma of both groups. In muscle, phage accumulation was maintained over time in all of the treated mice (Table 1). All tissues from the untreated animals were negative for bacteriophages.

### Transcytosis of Bacteriophages Across the *in vitro* Intestinal Barrier Model

**FIGURE 3:**
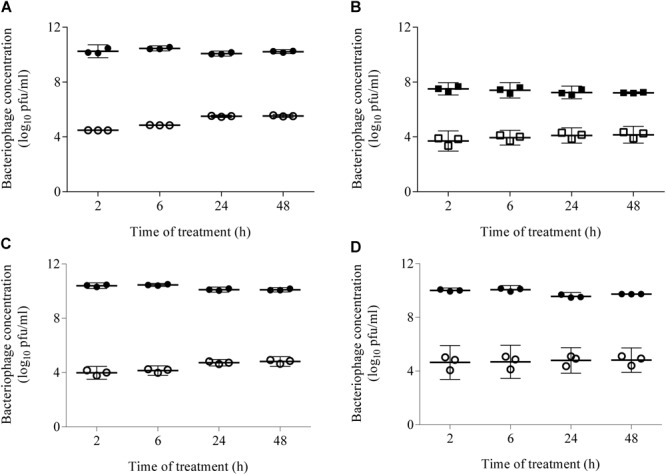
Transcytosis of non-encapsulated phage **(A–C)** and PPE **(D)** across an intestinal epithelial monolayer. The bacteriophage concentration was quantified 2, 6, 24, and 48 h post-inoculation by titration of the contents of the apical (black ●, ■) and basolateral (white ○, □) compartments. DMEM supplemented with FBS was used to study the non-encapsulated bacteriophages **(A,B)** and serum-free DMEM to compare the transcytosis of non-encapsulated phages **(C)** and PPE **(D)**. The concentrations applied in the apical chamber were 1 × 10^10^ pfu/mL (circles) and 1 × 10^7^ pfu/mL (squares). Scatter plots show the means; error bars represent the 95% confidence intervals. The phage concentration in the basolateral chamber is the sum of the values obtained at each time point.

The TEER of the intestinal epithelial cell monolayers was measured before and after (48 h) bacteriophage inoculation. All TEER values were >300 Ω/cm^2^, confirming the valid use of this method in the experiments. In addition, epithelial cell monolayer polarity and integrity were assessed by TEM, which revealed that the bacteriophages did not alter either the membrane stability of Caco-2 cells or the robustness of their tight junctions (Supplementary Figure S2). Transcytosis of the bacteriophages applied to the apical side of the intestinal epithelial cell monolayers was quantified at 2, 6, 24, and 48 h by plating the apical and basolateral contents onto LB5000 agar plates. The results showed that, after 2 and 48 h, bacteriophage transcytosis in DMEM containing FBS ranged from 4.5 ± 0.0 to 5.5 ± 0.1 log_10_ pfu/mL and from 3.7 ± 0.3 to 4.2 ± 0.3 log_10_ pfu/mL at starting concentrations of 1 × 10^10^ pfu/mL and 1 × 10^7^ pfu/mL, respectively (Figure 3). Because the amount of transcytosis was low, only the higher starting dose (1 × 10^10^ pfu/mL) was used in the PPE experiment. Furthermore, the experiments were conducted in serum-free DMEM because FBS caused liposomal aggregation (data not shown). Prior to the transcytosis experiments, we demonstrated that incubation of the PPE in DMEM at 37°C for 72 h did not modify either the titer or the percentage of encapsulation (around 46% of the applied dose). Thus, under this condition, the transcytosis of UAB_Phi20 phages was similar that described above for a starting dose of 1 × 10^10^ pfu/mL. Accordingly, bacteriophage encapsulation did not significantly modify transcytosis at either 2 or 48 h, based on values of 4.6 ± 0.5 and 4.8 ± 0.4 log_10_ pfu/mL, respectively (Figure 3). It should be noted that encapsulated bacteriophages were not found in the basolateral compartment at any of the sampling times. Furthermore, the percentage of encapsulated phages in the apical section did not change during the experiment, regardless of the initially applied concentration (data not shown).

**FIGURE 4:**
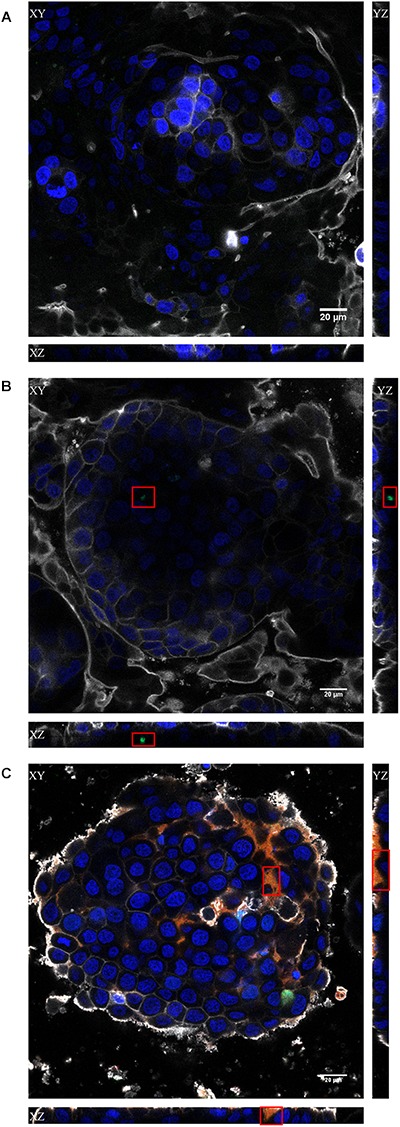
Confocal images of the *x, y*, and *z* scans of the *in vitro* Caco-2/HT29/Raji-B lymphocytes co-culture model. The results obtained with the non-treated **(A)**, bacteriophage-treated **(B)** and PPE **(C)** treated cultures at 2 h are shown. Cell nuclei were stained with Hoechst 33242 (blue), bacteriophages with SYBR gold (green), and liposomes with Vybrant Dil (red). The plasma membrane was stained with CellMask DeepRed (gray). Red squares indicate the regions where the stained phages **(B)** or PPE **(C)** were visualized. Scale bars, 20 μm.

**FIGURE 5:**
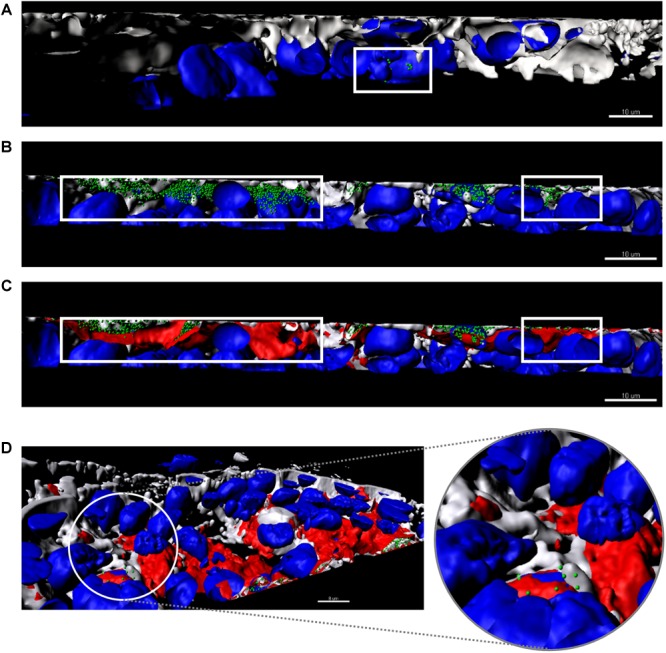
Three-dimensional images of a cross-section of the intestinal barrier model based on a Caco-2/HT29/Raji-B lymphocytes co-culture treated with bacteriophages **(A)** and PPE **(B–D)**. Cell nuclei were stained with Hoechst 33242 (blue), bacteriophages with SYBR gold (green), and liposomes with Vybrant Dil (red). The plasma membrane was stained with CellMask DeepRed (gray). **(A)** Non-encapsulated phages (green) are seen inside the cells. **(B,C)** Stained PPE in different phases. **(B)** Only encapsulated phages (green) are shown inside the cells (gray, membrane and blue, cell nuclei). **(C)** Merging of all labels with the liposomes (red) covering the encapsulated phages detailed in **(B)**. The white square indicates the non-encapsulated phages **(A)**, the encapsulated phages **(B)**, and liposome capsule **(C)**. **(D)** A detail of the basolateral side of the intestinal barrier (sited in the upper side of the image) shows the presence of liposomes (red) inside the cells. The enlarged image shows phages released from the liposomes. Scale bars, 8 μm.

To visualize the transcytosis of bacteriophages through the cell monolayer, bacteriophage UAB_Phi20 was fluorescently labeled using SYBR gold and encapsulated in liposomes labeled with Vybrant Dil. Both the labeled phages and the labeled liposomes containing labeled phages were incubated with intestinal cell monolayers for 2 h. Figure 4 shows the CLSM images depicting the fluorescent signals corresponding to the cells (Figure 4A), phages (Figure 4B), and PPE (Figure 4C) stratified across the barrier thickness, as revealed by the *x, y*, and *z* scans of the intestinal cell layer. In addition, three-dimensional images of a cross-section of the cell layer, created using the Imaris^®^ software, showed the merging of the different fluorophores used for staining the cell membrane, nucleus, phages, and liposomes (Figure 5). Non-encapsulated bacteriophages were seen inside the intestinal cells (Figure 5A) and phages from the labeled PPE were present at high density on the cell surface and inside the cells (Figure 5B–D). The latter observation reflected adherence of the liposomes containing the labeled phages, which was corroborated by the coincidence of the fluorescence emitted by the stained phages and liposomes. In addition, aggregation of the liposomes resulted in the formation of very large (μm) bacteriophage-containing masses (Figure 5C). These aggregates were mainly embedded in the cell membrane or remained on the cell surface, but some were also seen inside the cells (Figure 5C). Figure 5D shows the presence of liposomes inside the cells with a detail of the basolateral side of the cell culture (sited in the upper side of the image) and bacteriophages released from the liposomes are also seen in this figure.

## Discussion

The purpose of this work was to investigate the adherence of encapsulated phages in liposomes to the intestinal barrier and the ability of the phages to cross this barrier. The behavior of the encapsulated phages was studied in an athymic nude mouse model using a non-invasive methodology based on the visualization of fluorophore-labeled bacteriophages administered orally in a single dose. Although our original intent was to determine the biodistribution of encapsulated phages in chickens, technical problems related to the autofluorescence of the down of the chicks prevented the development of this model.

In the *in vivo* study, the emitted fluorescence provided a clear visualization of the bacteriophages in the mouse gastrointestinal tract (stomach and intestine) (Figure 1). *Ex vivo* analysis of the fluorescently labeled bacteriophages in organs removed from the mice further revealed an increase in the phage concentration in the stomach, but not in the intestine, 3 h after PPE administration (Figure 2C). Moreover, accumulation in the stomach was maintained for at least an additional 3 h, although the phage concentration decreased under all conditions (Figure 2C). The concentration of labeled bacteriophage in the organs examined *ex vivo* using conventional microbiological techniques confirmed the effect of encapsulation on phage accumulation in the stomach at both 3 and 6 h; that is, the absence of a decrease in the bacteriophage concentration (Table 1). While 50% of the phages present in the stomach were encapsulated, no encapsulated phages were detected in the intestine (Table 1). In a previous study in a chicken model, we showed that the cationic lipid mixture used in encapsulation protected the bacteriophages against the acidic pH of simulated gastric conditions and that encapsulated bacteriophages incubated with cecal contents were released from the capsules. While liposome-encapsulated phages were detected 72 h after the administration of a single dose to non-infected chickens, non-encapsulated bacteriophages were scarcely present (Colom et al., 2015). We thus hypothesized a high retention capacity of encapsulated bacteriophages in the stomach that enabled the slow delivery and continuous presence of the bacteriophages in the intestine, thereby contributing to prolonged and successful oral phage therapy. The *in vivo* and *ex vivo* data presented herein provide further support for this hypothesis.

Fluorophore-labeled bacteriophages could not be observed, either *in vivo* or *ex vivo*, in organs or tissues other than those above-mentioned, because the phage concentrations were below the limit of detection of the IVIS imaging system (∼7 log_10_ pfu/g). Further studies using a different fluorophore, a higher phage concentration, or a more powerful visualization system are therefore needed to improve phage detection. To our knowledge, the *ex vivo* or *in vivo* visualization of bacteriophages in living systems has been achieved in only a few studies and they were carried out using labeled/modified-phages to deliver pharmaceuticals by intravenous injection (Kelly et al., 2006; Kaźmierczak et al., 2014). Similar to our observations, the results of those studies demonstrated the utility of molecular imaging to track phages within living systems.

The presence and persistence of non-encapsulated phages in the liver, spleen, and kidney and in the blood have been described by several authors (Dabrowska et al., 2005; Górski and Weber-Dabrowska, 2005; Górski et al., 2006; Hamzeh-Mivehroud et al., 2008; Oliveira et al., 2009; Midddedzybrodzki et al., 2017). In this work, we asked whether encapsulated bacteriophages could cross the intestinal barrier to reach internal organs and tissues, as already observed for non-encapsulated bacteriophages. The liposome-encapsulated phages prepared for use in this work had a size of 341.6 ± 8.6 nm, a zeta potential of + 34 ± 5.0 mV, and a capsule formulation that included cholesterol and cholesteryl polyethylene glycol 600 Sebacate (Colom et al., 2015). Thus, the liposomes were of a size (<500 nm) that allowed their cellular uptake (Jung et al., 2000; Li et al., 2016; Neves et al., 2016) while the cholesterol or PEG improved their stability in the circulation, reducing the likelihood of their removal by the reticuloendothelial system (Bozzuto and Molinari, 2015; Sercombe et al., 2015). Moreover, the hydrophobicity and positive charge of the capsules favored their mucoadhesion, resulting in their enhanced cellular uptake and lymphatic delivery (Jung et al., 2000; Sercombe et al., 2015; Ahn and Park, 2016). Our *ex vivo* data showed that the number of phages that reached the liver, kidney, and even muscle within 6 h post-administration was significantly higher in mice administered non-encapsulated phages than in the PPE-treated mice (Table 1). Only in the spleen, at 3 h post-administration, was there a significant difference between the two groups of mice. This result suggested that the intestinal barrier is more easily crossed by non-encapsulated than by encapsulated phages. In addition, the lipid coat of the phages seemed to favor accumulation in certain organs and tissues, as suggested by the percentage of encapsulated phages present in the spleen (68.0 ± 8.6), liver (38.4 ± 6.3), and muscle (47.0 ± 7.4) of the mice 3 h after PPE administration. By contrast, no encapsulated phages were detected in the kidney and hardly any in the plasma (Table 1). To our knowledge, ours is the first study to track orally administered, liposome-encapsulated phages in different organs, although the detection of liposome-entrapped phages in the lungs, liver, kidney, and spleen of BALB/c mice after 6 h was previously reported (Singla et al., 2016). However, in that work, the phages were administered by intraperitoneal injection, which may account for the differences compared to our study.

The *in vivo* and *ex vivo* results obtained in our mouse model indicated the preferential accumulation of encapsulated bacteriophages in the stomach, at least during the first 6 h post-administration, and that after reaching the intestine they translocated to other organs and tissues. Translocation seems to be a rapid process because encapsulated phages were not found in the intestine as early as 3 h after their oral administration. However, the continued presence of encapsulated bacteriophages in the intestine cannot be ruled out because homogenization of the intestinal tissue in MgSO_4_, as performed in the *ex vivo* experiments, breaks the phage capsules in a process mediated by the intestinal bile salts of the host mice. Nonetheless, encapsulated bacteriophages were found in internal organs, including the spleen, liver, and muscle.

The ability of phages encapsulated in liposomes to overcome the intestinal barrier was further studied in an *in vitro* model based on the co-culture of Caco-2 and HT29 intestinal cells with Raji-B lymphocytes. This model approximates the intestinal barrier and allows comparisons of the permeation capacity of nanoparticles with the findings obtained in *in vivo* studies (Antunes et al., 2013). Our data showed that the presence of the phages did not affect the integrity of intestinal cell junctions (Supplementary Figure S2) and that bacteriophage transcytosis occurred at a low level (Figure 3A–C). However, CLSM revealed the presence of bacteriophages in the cytosol of intestinal cells (Figure 4B,C). It should also be noted that with a phage starting dose of 1 × 10^7^ pfu/mL our transcytosis results agreed with those obtained using P22 in MDCK cells (Nguyen et al., 2017). Thus, the model used in this study can be employed in other investigations of phage transcytosis.

The percentage of bacteriophages that crossed the membrane following PPE administration was also low (Figure 3D) and encapsulated bacteriophages could not be detected using conventional microbiological methods (data not shown). Nevertheless, in this model, liposome capsules were seen adhering to the cell surface as aggregates, embedded in the cell membrane, and even present inside the cells (Figure 5C,D). These observations and the detection of bacteriophages inside the liposomes together suggest that liposomes protect phages against their elimination from the intestinal tract by excretion. Following the eventual release of these phage from the liposomes, they may undergo transcytosis. Unfortunately, however, these results could not be compared with those obtained in our *in vivo* model because the Transwell membrane obviously does not reproduce the open system of the intestinal tract, among other differences.

Taken together, our results suggest that orally administered liposome-encapsulated phages remain in the stomach and that encapsulation protects the phages until their release. Moreover, when encapsulated phages reach the intestine, adherence to the intestinal wall temporarily protects them from both the action of bile salts and elimination via excretion. We hypothesize that liposomes and the phages they contain most likely reach internal organs through the duodenum, the segment of the small intestine with the highest absorption rate (Oliveira et al., 2009; Li et al., 2016; Van Den Abeele et al., 2017). This region contains M-cells, whose characteristics (high transcytosis capacity, few lysosomes, thinner mucous glycocalyx) (Kou et al., 2013; He et al., 2018) favor the access, uptake, and transport of both the positively charged liposome capsules and the phages to the underlying lymphoid tissues, albeit by different mechanisms (des Rieux et al., 2006; Kou et al., 2013; Li et al., 2016; Yu et al., 2016), thus enhancing the systemic availability of the bacteriophages. The accumulation of encapsulated phages in the stomach and their adherence to the intestinal wall would explain our previous observations (Colom et al., 2015) of the greater long-term efficacy of phage therapy using liposome-encapsulated phages.

## Conclusion

This study is the first to demonstrate the biodistribution and transcytosis of orally administered, liposome-encapsulated bacteriophages. In a murine model based on a non-invasive methodology, labeled phages were visualized in the mouse stomach and intestine. Moreover, conventional culture methods revealed the additional presence of liposome-encapsulated phages in the stomach and other internal organs, including the spleen and liver, and in muscle. The adherence of liposome-containing phages to human intestinal cells, their embedding within the cells, and the transcytosis of the phages was evidenced by CLSM. Our results contribute to the development of treatment options based on oral phage therapy. Nonetheless, further efforts are required to improve bacteriophage labeling or *in vivo* imaging in order to track the dissemination of liposome-encapsulated phages.

## Ethics Statement

The *in vivo* experiments were performed by in the ICTS “NANBIOSIS,” specifically, by the CIBER-BBN’s *in vivo* experimental platform of the Functional Validation & Preclinical Research (FVPR) area (http://www.nanbiosis.es/portfolio/u20-in-vivo-experimental-platform/) and Laboratory Animal Service (LAS) of Vall d’Hebron Institut de Recerca (VHIR; Barcelona). The animals were treated in agreement with the guidelines of the Ethics Commission [Comité Ético de Experimentación Animal (CEEA)] of the VHIR, Barcelona. The study was approved and assigned the authorization number 69/15.

## Author Contributions

JO executed most of the bacteriophage experiments, including the *ex vivo* experiments using conventional microbiology methods, phage labeling, and encapsulation, intestinal cell culture, and statistical and imaging analysis. AG-R assisted in establishing and maintaining the intestinal cell cultures. MC-S assisted in bacteriophage encapsulation. DM and RM reviewed the manuscript. PC and ML contributed to the design of the study, analyses of the results, coordination of the study, and drafting the manuscript. All authors read and approved the final version of the manuscript.

## Conflict of Interest Statement

PC and ML are the inventors in patent application EP2750519. The remaining authors declare that the research was conducted in the absence of any commercial or financial relationships that could be construed as a potential conflict of interest.

## Footnotes

1 http://www.nanbiosis.es/portfolio/u20-in-vivo-experimental-platform/

## Supplementary Material

FIGURE S1 Cryo-TEM image of liposome-encapsulated UAB_Phi20; **(B)** 3D confocal images of SYBR gold-labeled UAB_Phi20 encapsulated into fluorescent Dil-labeled liposome (red). 3D image of liposome surface is shown on the left and its cross-section images on the right. Scale bars, 5 μm.

FIGURE S2 Epithelial cell monolayer TEM images after 48 h of incubation with the non-encapsulated bacteriophage. Tight junctions are indicated by a white ellipse. Scale bars are shown in the images.

TABLE S1 Determination of the encapsulation yield (%) of UAB_Phi20 after three independent experiments.
