# Supplementary figures and images for: Biodistribution of Liposome-Encapsulated Bacteriophages and Their Transcytosis During Oral Phage Therapy
Source: Front Microbiol. 2019 Apr 4;10:689. doi: 10.3389/fmicb.2019.00689 (PMC6458305; doi:10.3389/fmicb.2019.00689)

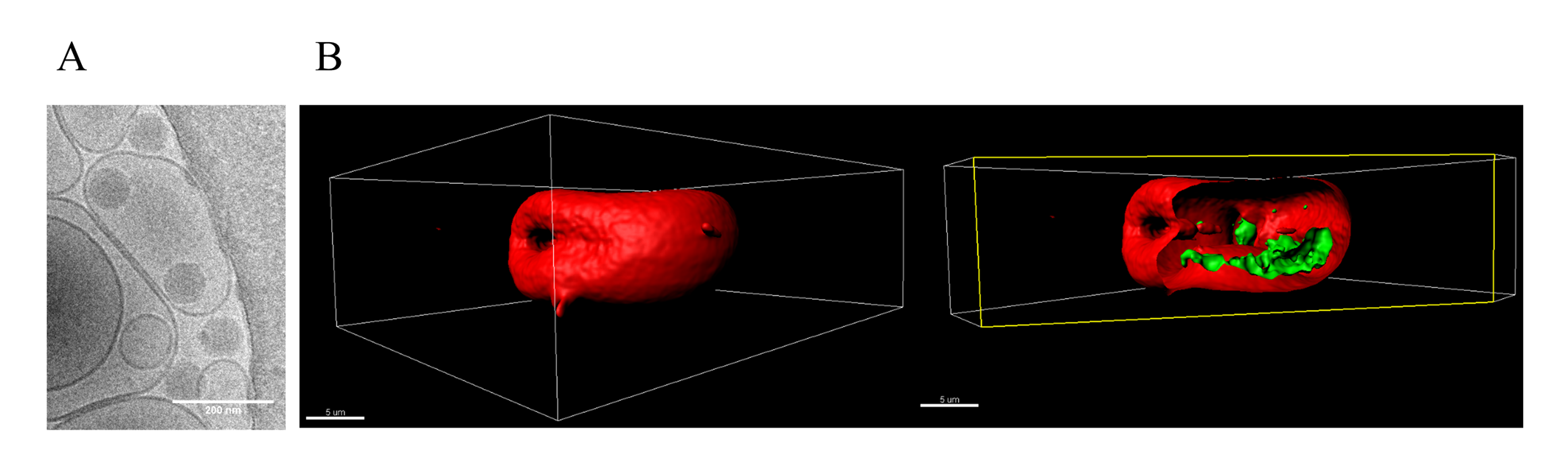

Supplement: FIGURE S1 — Cryo-TEM image of liposome-encapsulated UAB_Phi20; (B) 3D confocal images of SYBR gold-labeled UAB_Phi20 encapsulated into fluorescent Dil-labeled liposome (red). 3D image of liposome surface is shown on the left and its cross-section images on the right. Scale bars, 5 μm. [file Image_1.TIF]

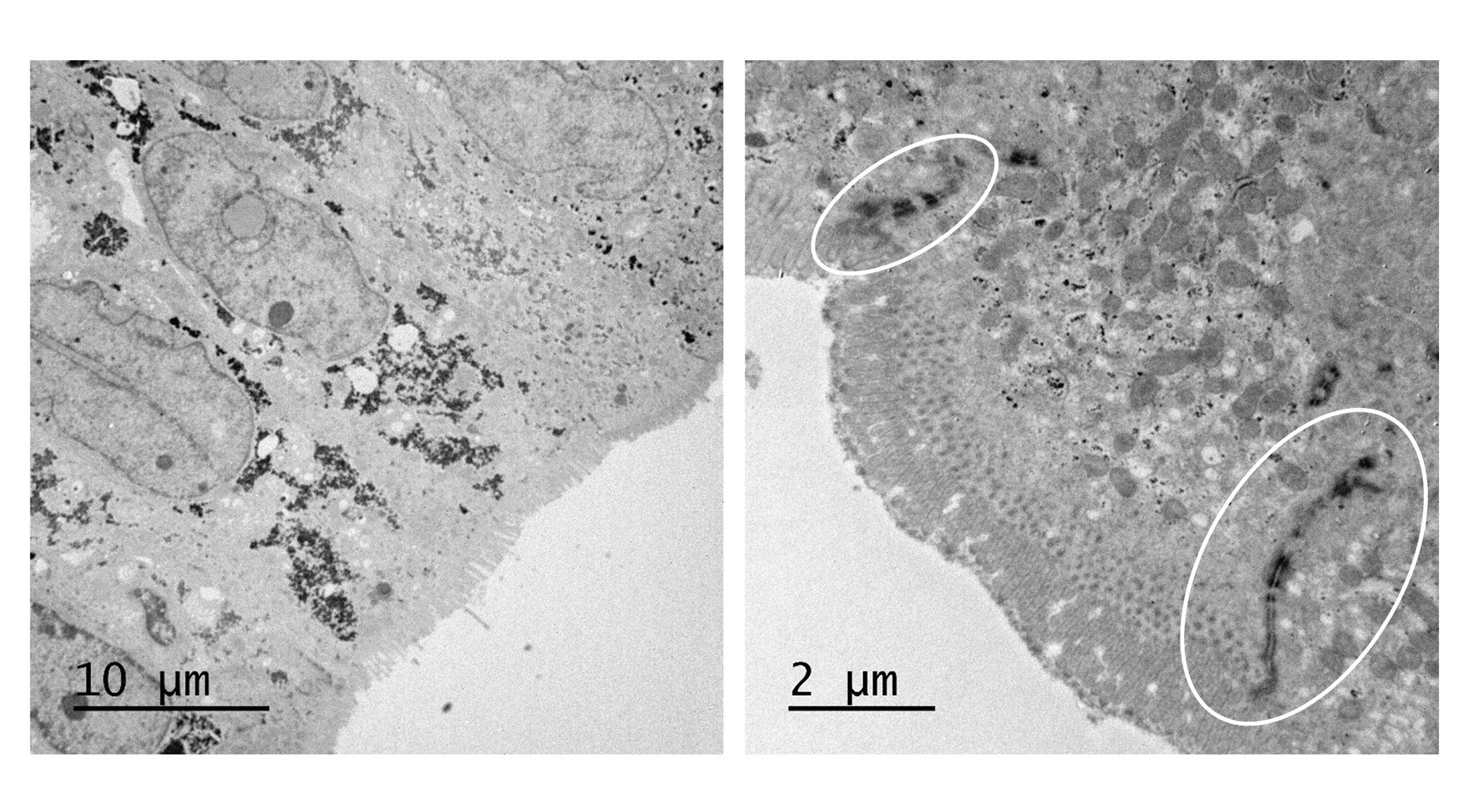

Supplement: FIGURE S2 — Epithelial cell monolayer TEM images after 48 h of incubation with the non-encapsulated bacteriophage. Tight junctions are indicated by a white ellipse. Scale bars are shown in the images. [file Image_2.TIF]
