# Supplementary material for: Biodistribution of Liposome-Encapsulated Bacteriophages and Their Transcytosis During Oral Phage Therapy
Source: Front Microbiol. 2019 Apr 4;10:689. doi: 10.3389/fmicb.2019.00689 (PMC6458305; doi:10.3389/fmicb.2019.00689)
Supplement: TABLE S1 — Determination of the encapsulation yield (%) of UAB_Phi20 after three independent experiments. [file Table_1.pdf]

Supplementary Material

Biodistribution of liposome-encapsulated bacteriophages and their transcytosis during oral phage therapy

Jennifer Otero[1], Alba García[1], Mari Cano-Sarabia[2], Daniel Maspoch[2,3], Ricard Marcos[1,4], Pilar Cortés[1*], Montserrat Llagostera[1]

***Correspondence**: mariapilar.cortes@uab.cat

1. Supplementary Tables

Supplemental material includes one supplementary Table 1.

Supplementary Table S1. Determination of the encapsulation yield (%) of UAB_Phi20 after three independent experiments.

Experiments	Bacteriophage concentration (pfu/mL)[a]		Encapsulation yield (%)		
	Free	Total[b]	Individual	Mean	SD
1	7.1×10^{10}	1.3×10^{11}	44.5		
2	9.9×10^{10}	1.8×10^{11}	45.3	45.7	1.5
3	3.4×10^{10}	6.5×10^{10}	47.4		

a Each value represents the average of three replicate plates.

b Values obtained after treatment with bile salts.
